# Misconfigured striatal connectivity profiles in smokers

**DOI:** 10.1038/s41386-022-01366-6

**Published:** 2022-06-25

**Authors:** Cole Korponay, Elliot A. Stein, Thomas J. Ross

**Affiliations:** 1 Basic Neuroscience Division, McLean Hospital, Belmont, MA USA; 2 Neuroimaging Research Branch, Intramural Research Program, National Institute on Drug Abuse, Baltimore, MD USA

**Keywords:** Addiction, Synaptic transmission

## Abstract

Dysregulation of frontal cortical inputs to the striatum is foundational in the neural basis of substance use disorder (SUD). Neuroanatomical and electrophysiological data increasingly show that striatal nodes receive appreciable input from numerous cortical areas, and that the combinational properties of these multivariate “connectivity profiles” play a predominant role in shaping striatal activity and function. Yet, how abnormal configuration of striatal connectivity profiles might contribute to SUD is unknown. Here, we implemented a novel “connectivity profile analysis” (CPA) approach using resting-state functional connectivity data to facilitate detection of different types of connectivity profile “misconfiguration” that may reflect distinct forms of aberrant circuit plasticity in SUD. We examined 46 nicotine-dependent smokers and 33 non-smokers and showed that both dorsal striatum (DS) and ventral striatum (VS) connectivity profiles with frontal cortex were misconfigured in smokers—but in doubly distinct fashions. DS misconfigurations were stable across sated and acute abstinent states (indicative of a “trait” circuit adaptation) whereas VS misconfigurations emerged only during acute abstinence (indicative of a “state” circuit adaptation). Moreover, DS misconfigurations involved abnormal connection strength rank order arrangement, whereas VS misconfigurations involved abnormal aggregate strength. We found that caudal ventral putamen in smokers uniquely displayed multiple types of connectivity profile misconfiguration, whose interactive magnitude was linked to dependence severity, and that VS misconfiguration magnitude correlated positively with withdrawal severity during acute abstinence. Findings underscore the potential for approaches that more aptly model the neurobiological composition of corticostriatal circuits to yield deeper insights into the neural basis of SUD.

## Introduction

Nicotine dependence remains the largest cause of preventable death in the United States [1]. A primary obstacle to quitting is the generally negative phenomenology of the Nicotine Withdrawal Syndrome (NWS) brought on following nicotine’s acute absence, which peaks in the immediate days following abstinence. Mounting evidence from human neuroimaging studies indicates that much of the NWS phenomenology that arises can be traced to alterations in specific brain circuits and networks [2–7]. As such, advancing the understanding of how brain circuitry reconfigures during this critical time window, including how “state” reconfigurations during acute abstinence interact with underlying “trait” reconfigurations associated with long-term nicotine use, has the potential to drive more efficacious circuit-guided treatment approaches.

Among the most extensively implicated circuits in the pathology of nicotine dependence, and SUD more broadly, is the corticostriatal system. This circuitry is characterized by monosynaptic projections that transmit information processed within the frontal cortex to the striatum [8], the major input nucleus to the basal ganglia. The ventral striatum and its frontal cortical connections have established roles in motivational and affective processes and are implicated in aberrant reward processing and craving in SUD [9–11]. Meanwhile, the dorsal striatum is central to the execution of goal-directed and stimulus-response behaviors that become dysregulated in addiction [12**–**15].

Studies leveraging resting-state functional connectivity (rsFC), a non-invasive measure of the coherence of spontaneous activity between different brain areas [16], have repeatedly demonstrated dysregulated connectivity in both frontal-ventral striatum (VS) and frontal-dorsal striatum (DS) circuits in smokers [4, 7, 17–19]. Yet, though prior work has mostly focused on the properties of “A-to-B” cortex-striatum node-pair connections, neuroanatomical tract-tracing studies have long shown the individual striatal nodes receive input from numerous different cortical areas [20, 21]. Recent retrograde tracing data has cast even clearer light on 1) the comparatively modest input that any given cortical region contributes to a striatal node, and 2) the large number of cortical regions that contribute input of non-negligible strength to a given striatal node [22**–**24]. For instance, across nine nodes examined in different regions of the macaque striatum [22, 23], each node’s strongest frontal cortical input region accounted for, on average, only 27.6% of its total frontal input; an average of 14.7 different regions contributed to the remaining ~75% of frontal input. This complements electrophysiological data demonstrating that striatal projection neurons require coordinated input from multiple afferents to drive sufficient depolarization for action potential generation [8, 25, 26].

If the net activity of striatal nodes is shaped more by combinational features of their multivariate “connectivity profiles” than by any individual cortical afferent, methods designed to detect “misconfigurations” in the connectivity profiles themselves may provide novel insights about the kinds and locations of circuit alterations most central to SUD pathology. As such, here we introduce a connectivity profile analysis (CPA) approach for quantifying, spatially localizing, and statistically assessing connectivity profile misconfiguration using null modeling [27]. Since a node’s connectivity profile could be altered along several different dimensions—each of which may reflect a distinct form of circuit plasticity—we defined and computed three complementary metrics for the novel measurement of connectivity profile misconfiguration: aggregate divergence, rank order misarrangement, and entropy shift. By leveraging an approach that better reflects the known neuroanatomical architecture of corticostriatal circuits, we aimed to gain deeper insights into the mechanisms of circuit dysfunction in addiction and about the sites with the greatest potential for localized therapeutic targeting in the future.

## Methods and materials

### Participants

#### Empirical Sample

Participants were right-handed, aged 18–55 years, free of active drug or alcohol abuse/dependence (other than nicotine dependence in smokers), reporting no current psychiatric or neurological disorders, and presenting no contraindications for magnetic resonance imaging (MRI). Fifty-four current smokers and 35 non-smoking controls completed all experimental procedures. Following preprocessing, data from eight smokers and two non-smokers were excluded due to excessive head motion (see below). Therefore, the final empirical sample for analysis consisted of 46 smokers (27 male; mean age 38.20 ± 11.99, mean years of education 13.00 ± 1.92) and 33 non-smokers (21 male; mean age 36.48 ± 9.57, mean years of education 13.55 ± 1.95). Groups did not differ in age, *t*(77) = 0.719, *p* = 0.474, sex, *X*^2^(1,79) = 0.197, *p* = 0.657, or years of education, *t*(77) = 1.236, *p* = 0.220. Written informed consent was obtained in accordance with the National Institute on Drug Abuse, Intramural Research Program Institutional Review Board.

#### Normative sample

The CPA procedure requires the computation of a normative distribution for each connectivity profile misconfiguration metric, to facilitate statistical assessment of empirically observed values. To do so, a normative sample from the Human Connectome Project (HCP) was curated to match the empirical sample (i.e., sample size, demographics, head motion) except for the presence of the group difference of interest in the empirical sample (i.e., smoking status). This allows the observation of empirically measured values that fall significantly outside the distribution of normative values to be attributed to the group difference of interest in the empirical sample. Procedures used to curate this matched normative sample are described in the Supplementary Methods.

### Experimental design

Each non-smoker completed one MRI scanning session, and each smoker completed two MRI scanning sessions: one immediately following ad lib smoking, and the second during acute abstinence ~48 h after smoking the last cigarette prior to scanning. Since these data were part of an extended treatment protocol, the order of the two scans was fixed, with the sated scan preceding the abstinent scan by an average of 67 days (median 28 days). Further details on biochemical verification of abstinence, experimental protocol, and subject exclusion criteria are provided in the Supplemental Materials.

### MRI data acquisition

Whole-brain echo-planar images for 57 subjects (26 non-smokers and 31 smokers) were acquired on a 3T Siemens Trio scanner (Erlangen, Germany) using a 12-channel head coil, and images for 22 subjects (7 non-smokers and 15 smokers) were acquired on a Prisma using a 20-channel head coil. Both scanners used the same acquisition parameters (see Supplementary Materials for details), and both involved 8-min eyes-open scans with participants instructed to let their minds wander. The proportion of subjects scanned on each scanner did not differ between the non-smoker and smoker groups, *X*^2^(1,79) = 1.24, *p* = 0.265. Furthermore, after preprocessing with the same pipeline, no significant differences in connectivity Z-score maps were observed across subjects in relation to which scanner was used. As such, images from both scanners were analyzed together to maximize statistical power.

### Resting-state fMRI preprocessing

Preprocessing was performed using FMRIPREP version 20.2.1. A detailed description of these preprocessing steps is included in the Supplementary Methods. Further preprocessing included spatial blurring with a 6-mm full-width half-maximum Gaussian kernel and temporal filtering (0.01 < *f* < 0.1 Hz). To control for subject head motion, volumes were censored for framewise motion displacement (i.e., volume to volume movement) [28, 29]. The following frames were censored: frames with a framewise displacement (FD) > 0.5 mm, frames preceding those with a FD > 0.5 mm, and the first three frames of each scan. Subjects with more than 25% of frames censored were excluded from analysis. Eight smokers and two non-smokers were excluded from final analyses due to these head motion criteria.

rsFC between each striatal voxel and each frontal cortical seed (ROI) (see Supplementary Methods) was assessed using the mean resting-state BOLD time series from each ROI extracted from each participant, which was then included in a GLM with 17 additional regressors of no interest: six motion parameters (three translations and three rotations) obtained from the rigid-body alignment of EPI volumes and their six temporal derivatives; the mean time series extracted from white matter; the mean times series extracted from CSF; and a second-order polynomial to model baseline signal and slow drift. The output of *r* values from the GLM was converted to Z-scores using Fisher’s *r*-to-Z transformation. Finally, given group differences in head-motion (Fig. S2), framewise displacement-adjusted Z-score maps were computed and served as the inputs to all main analyses (see Supplementary Methods for details and procedure).

### Resting-state fMRI analysis

As the foundation for our analytic pipeline, we first established a frontal cortex connectivity profile (“fingerprint”) [30] for each voxel in the striatum. Each fingerprint quantified the strength of rsFC between a given striatal voxel and 30 (15 ipsilateral, 15 contralateral) frontal cortical subregions (“targets”) [31], and was encoded by 30 voxel-wise striatal Z-score maps (one for each ROI) for each subject. Subject-level fingerprints were then used to create three sets of group-level fingerprints—one averaged set each for sated smokers, abstinent smokers, and non-smokers.

Subsequently, we adopted three complementary metrics for evaluating distribution properties [30, 32, 33] and applied them in a novel manner to quantify how voxel-wise connectivity profiles differed along distinct dimensions between the subject groups. The first, which we termed “aggregate divergence”, measured the absolute cumulative magnitude by which all matched connections across two connectivity profiles differed. The second, which we termed “rank order misarrangement”, measured how the order of strongest to weakest connections differed between two connectivity profiles. The third, which we termed “entropy shift”, measured differences in how connectivity profile strength was concentrated in a few connections versus distributed appreciably across many connections. Computations were carried out using custom MATLAB scripts (version R2020b, publicly accessible at https://github.com/ckorponay/Connectivity-Profile-Misconfiguration). Computation procedures are detailed in the Supplementary Methods.

#### Determination of significance thresholds for group-level analysis

To determine whether empirically observed connectivity profile differences were large enough to constitute statistically significant “misconfigurations”, we established a normative distribution of each of the three connectivity profile difference metrics via repeated permutation of the matched “null” data from the HCP sample, which was also FD-adjusted in the same manner as the empirical sample (details of procedure in Supplementary Methods). Voxel-wise *p* < 0.001 thresholds for each metric were determined as: aggregate divergence ≥ 2.675; rank order misarrangement ≥ 160; entropy shift ≥ 0.045 (Fig. S5a–c). Corrected thresholding was set using 3dClustSim (AFNI 20.1.14) at *p* < 0.001 uncorrected and *k* > 7 to yield a *p*_FWE_ < 0.05.

#### Within-group analysis

To identify striatal areas where connectivity profiles undergo significant within-smoker change following the transition from satiety to 48-h abstinence, we first computed subject-level maps for each of the three connectivity profile difference metrics. This was done by replacing the smoker-average voxel-wise Z-score maps (as described above) with the individual subject maps and evaluating each smoker’s connectivity profiles (in each state) with respect to the non-smoker group-average connectivity profiles. Then, for each of the three metrics, we performed a voxel-wise paired *t*-test comparing the maps of smokers in the sated and 48-h abstinent states.

#### Connectivity profile misconfiguration, dependence severity, and withdrawal symptomology

Lastly, we sought to determine whether interindividual variance in smokers’ connectivity profile properties was associated with differences in dependency severity, as measured by the Fagerstrom Test for Nicotine Dependence (FTND) [34], and withdrawal symptomology during acute abstinence, as measured by the Wisconsin Withdrawal Scale [35]. Controlling for age, sex, and years of education, we used linear regression to assess relationships between these metrics and subject-level connectivity profile difference metrics.

## Results

### Aggregate divergence

**Fig. 1:**
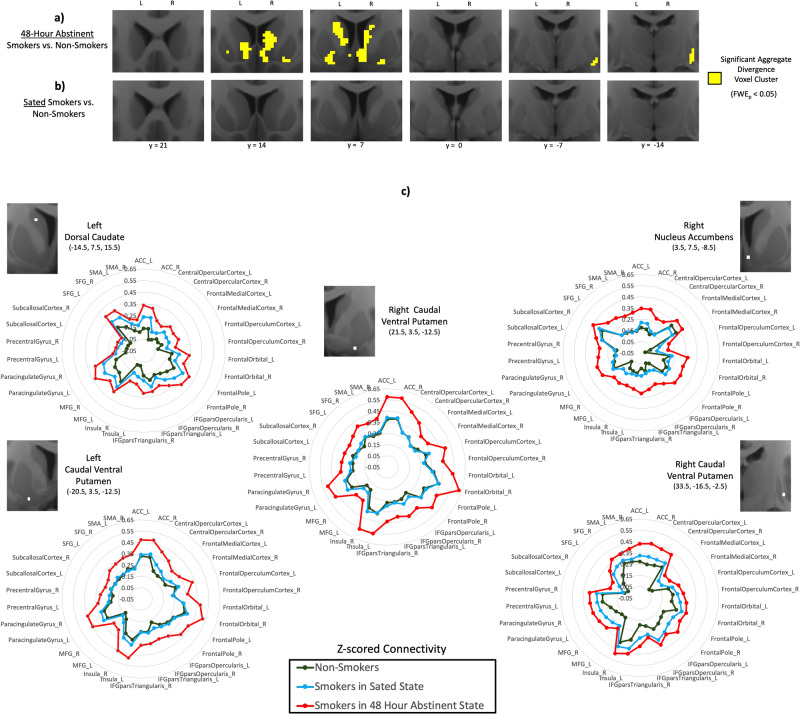
Aggregate divergence of striatal connectivity profiles in smokers relative to non-smokers. **a** Significant aggregate divergence (yellow) emerges in the medial and ventral striatum in smokers after 48 hours of verified abstinence, but **b** no aggregate divergence is evident when smokers are in a nicotine-sated state. **c** The connectivity profiles (“fingerprints”) of non-smokers (dark green), sated smokers (blue), and acutely abstinent smokers (red) at the striatal sites of peak aggregate divergence (Table S1) in acutely abstinent smokers. Distance of each point from center of plot denotes Z-scored functional connectivity strength between striatal site and labeled frontal cortical ROI. Gap between red line and green line illustrates aggregate divergence of the connectivity profile in acute abstinence.

After 2 days of verified abstinence, large sections of the right and left medial and ventral striatum in smokers displayed significant aggregate divergence relative to non-smokers (Fig. 1a). The sites of maximum aggregate divergence were in the right nucleus accumbens, bilateral caudal ventral putamen, and left dorsal caudate (Table S1). In contrast, we did not identify any evidence of significant aggregate divergence in these same smokers when in the nicotine-sated state (Fig. 1b).

Examination of the FC fingerprint of each group at each of the five peak coordinates (polar plots in Fig. 1c) revealed that striatal FC with all frontal cortical ROIs was larger in abstinent smokers relative to both sated smokers and non-smokers. Within smokers, a significant increase in aggregate divergence following the transition from nicotine satiety to 48-h abstinence was observed only in the left nucleus accumbens (*k* = 11, peak-level *t* = 3.75 at −6, 6, −12) (Fig. S6).

### Rank order misarrangement

**Fig. 2:**
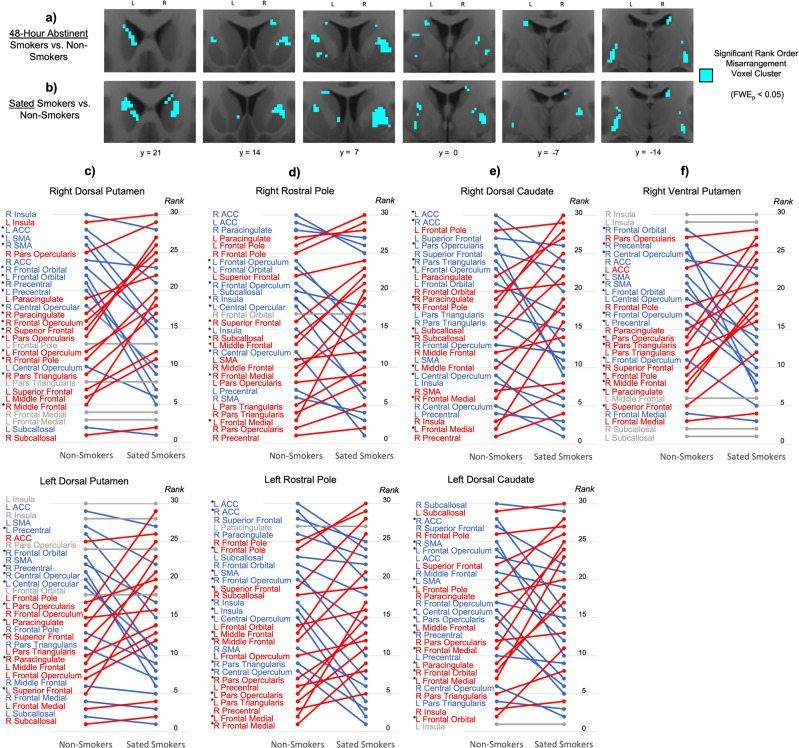
Rank order rearrangement of striatal connectivity profiles in smokers relative to non-smokers. Significant rank order rearrangement relative to non-smokers is present in smokers in both the **a** acutely abstinent state and **b** nicotine-sated state in the dorsal and lateral striatum (blue). **c**–**f** At the striatal sites where rank order significantly differed between non-smokers and sated smokers (Table S2), illustrations of how the rank order arrangement of non-smokers (left) differed from that of sated smokers (right). Cortical ROIs at the top (i.e., with high ranks) indicate those with stronger connectivity with the striatal site, while cortical ROIs at the bottom (i.e., with low ranks) indicate those with weaker connectivity with the striatal site. Red indicates cortical ROIs whose rank was higher in sated smokers than in non-smokers, while blue indicates cortical ROIs whose rank was lower in sated smokers than in non-smokers. Gray indicates cortical ROIs whose rank was the same in both groups. Star denotes cortical ROIs whose rank order difference between non-smokers and sated smokers was statistically significant (*p* < 0.001, rank order difference > 5).

Significant rank order misarrangement was identified in the right and left dorsal and lateral striatum of smokers in both the acutely abstinent (Fig. 2a) and nicotine-sated state (Fig. 2b). 69.7% of voxels that displayed significant rank order misarrangement during acute abstinence also displayed significant rank order misarrangement during satiety (Fig. S7), underscoring the constancy of rank order misarrangement in smokers across states. Moreover, within smokers, no significant changes in rank order arrangement were identified following the transition from satiety to 48-hour abstinence, further supporting the “trait” nature of this configuration difference. Sites of maximum rank order misarrangement during both satiety and abstinence included the right rostral dorsal putamen, right caudal ventral putamen, and left caudal dorsal putamen (Table S2).

Within both the right and left dorsal putamen clusters, connectivity strength rank was significantly lower in sated smokers compared to non-smokers for right precentral gyrus, left precentral gyrus, and right frontal orbital cortex (Fig. 2c). Conversely, rank was significantly larger in sated smokers compared to non-smokers for right superior frontal gyrus and left inferior frontal gyrus pars opercularis (Fig. 2c). Collectively, whereas dorsal putamen connectivity in non-smokers is stronger with motor and limbic frontal areas than with more cognitive frontal areas, rank order arrangement in sated smokers is swapped such that dorsal putamen connectivity is stronger with cognitive frontal areas than with motor and limbic frontal areas.

Within the right and left rostral pole clusters, connectivity strength rank was significantly greater in sated smokers compared to non-smokers for bilateral frontal medial cortex, but significantly lower in sated smokers for bilateral insula (Fig. 2d). The right and left dorsal caudate clusters were both characterized by significantly greater connectivity strength rank in sated smokers for left frontal medial cortex and left middle frontal gyrus, and significantly lower rank in sated smokers for right ACC, left inferior frontal gyrus pars opercularis, and left frontal opercular cortex (Fig. 2e). The right ventral putamen cluster was characterized by significantly lower connectivity rank in sated smokers with bilateral motor and premotor cortices and significantly greater connectivity rank with bilateral inferior and superior frontal gyri (Fig. 2f).

### Entropy shift

Evidence for significant entropy shift of striatal connectivity profiles in smokers compared to non-smokers was minimal (Table S3), with a handful of small clusters displaying entropy shift primarily in the acute abstinence state. Within smokers, entropy shift relative to non-smokers was significantly greater during acute abstinence than during satiety in the left rostral putamen (*k* = 16, peak-level *t* = 4.20 at −16, 12, −4) and left caudal caudate (*k* = 10, peak-level *t* = 4.16 at −10, 2, 18) (Fig. S6).

### Spatial segregation of different types of connectivity profile misconfiguration

**Fig. 3:**
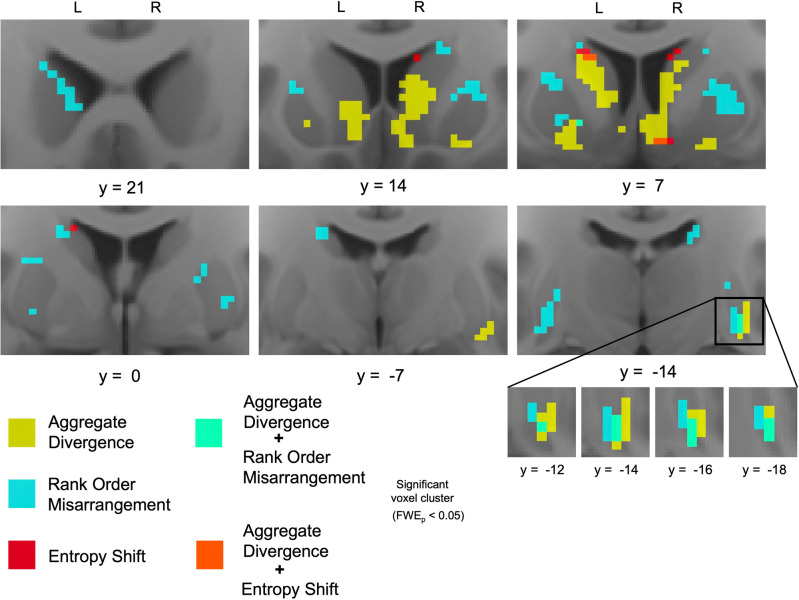
Spatial segregation of connectivity profile misconfiguration types. In smokers after 48-h abstinence (compared to non-smokers), spatial segregation of aggregate divergence (yellow) and rank order rearrangement (blue), and their unique co-occurrence in the right caudal ventral putamen (turquoise).

In acutely abstinent smokers (compared to non-smokers), striatal areas whose connectivity profiles displayed significant aggregate divergence, rank order misarrangement, and entropy shift were largely non-overlapping (Fig. 3). Of all identified voxels with significant aggregate divergence in acutely abstinence smokers, only 2.83% also displayed significant rank order misarrangement and only 1.51% also displayed significant entropy shift. Likewise, of all identified voxels with significant rank order misarrangement in acutely abstinent smokers, only 4.41% also displayed significant aggregate divergence and only 0.59% also displayed significant entropy shift. Spatially, this was reflected in a ventromedial/dorsolateral segregation, wherein significant aggregate divergence was primarily observed in medial and ventral striatum, while significant rank order misarrangement was primarily observed in lateral and dorsal striatum (Fig. 3).

### Focal spatial overlap in the right caudal ventral putamen

**Fig. 4:**
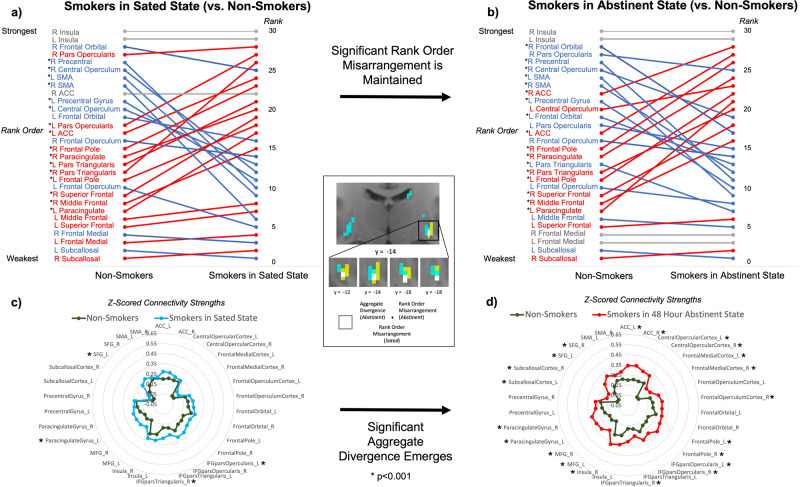
Multiple forms of connectivity profile reconfiguration in the right caudal ventral putamen (white voxels in center panel). Significant rank order rearrangement in smokers relative to non-smokers is evident during (**a**) nicotine satiety and is maintained following (**b**) 48-h abstinence. Significant aggregate divergence in smokers relative to non-smokers is not evident during (**c**) nicotine satiety but emerges after (**d**) 48-h abstinence.

However, a cluster of size *k* = 9 in right caudal ventral putamen uniquely displayed both significant aggregate divergence and significant rank order misarrangement in acutely abstinent smokers (Fig. 3). Moreover, six contiguous voxels within this cluster also displayed significant rank order misarrangement in sated smokers. We explored this unique *k* = 6 cluster in depth to determine precisely how these multiple types of connectivity profile misconfiguration manifested (Fig. 4). In smokers during satiety, the connectivity strength rank of motor and premotor control areas in the ventral putamen is significantly lower compared to non-smokers, while the rank of cognitive processing areas is significantly higher. This results in a right caudal ventral putamen connectivity profile in sated smokers where the rank order of motor and cognitive frontal area connectivity strengths is swapped compared to non-smokers (Fig. 4a). This swapped rank order arrangement is maintained in smokers following the transition from satiety to 48-h abstinence (Fig. 4b). However, though similar to non-smokers in aggregate connectivity magnitude during satiety (Fig. 4c), this rearranged connectivity profile also becomes significantly greater in aggregate strength during acute abstinence (Fig. 4d). This is driven by the emergence of significantly greater connectivity of right caudal ventral putamen with cognitive frontal areas—but not with motor or premotor frontal areas—compared to non-smokers.

### Connectivity profile misconfiguration, dependence severity, and withdrawal symptomology

We next explored whether the interactive magnitude of aggregate divergence and rank order misarrangement in the *k* = 6 right caudal ventral putamen cluster following the transition from satiety to acute abstinence was related to the severity of nicotine dependence and withdrawal symptomology across smokers. Controlling for age, gender, and education, as well as the main effects of aggregate divergence and rank order misarrangement, the interactive magnitude of these two connectivity profile misconfigurations was significantly negatively associated with dependence severity indexed by the FTND, *t*(37) = −2.510, *p* = 0.017. Low magnitudes of both misconfiguration types (i.e., connectivity profiles resembling non-smokers) and high magnitudes of both misconfiguration types predicted low FTND scores (Fig. S8). Conversely, a high magnitude of one misconfiguration type but a low magnitude of the other predicted high FTND scores. This was the only factor in the model significantly associated with dependence severity other than age, *t*(37) = 3.555, *p* = 0.001. Notably, separate tests of the main effect of aggregate divergence, *t*(39) = 0.480, *p* = 0.634, and of rank order misarrangement, *t*(39) = −0.182, *p* = 0.856, were not significant. See Supplementary Results for analysis of the relationship between connectivity profile misconfigurations and withdrawal symptomology.

## Discussion

We leverage a novel approach to model and quantify aberrance in understudied yet important units of corticostriatal circuit organization—the multipronged connectivity profiles of striatal loci with the subregions of frontal cortex. In doing so, we find that striatal connectivity profiles with frontal cortex are “misconfigured” in smokers. These misconfigurations manifest in different forms (aggregate divergence versus rank order misarrangement) and in different conditions (only during acute abstinence (state) versus stably across abstinence and satiety (trait)) in the ventral and dorsal striatum, respectively. Moreover, the magnitudes of connectivity profile misconfiguration in right caudal ventral putamen and left nucleus accumbens are linked to nicotine dependence and withdrawal severity, respectively. Foremost, these results suggest that SUD pathology need not necessarily derive from significant aberrance to individual frontostriatal connections, as observed in previous studies [7, 17, 36]. Aberrance in the configuration of striatal connectivity profiles—driven by the accumulation of small alterations to individual frontostriatal connections that in aggregate are substantial—appears to be another such neural foundation.

Importantly, we demonstrate that connectivity profile misconfigurations in the striatum manifest in several different and largely independent forms, as indexed by the metrics of aggregate divergence, rank order misarrangement, and entropy shift (though evidence for entropy shift in this study was minimal). Fewer than 5% of all striatal voxels that displayed significant connectivity profile misconfiguration in one form also displayed significant misconfiguration in another form. This supports the idea that each type of misconfiguration may reflect a distinct form of neurobiological plasticity, which may each relate to a separate alteration in the node’s activity and function. For example, appreciable increases (or decreases) to connection strengths throughout the connectivity profile—indexed by aggregate divergence—may upregulate (or downregulate) the node’s responsiveness to marginal input and its overall level of activity [37, 38]. Separately, the reshuffling of which connections are stronger and weaker—indexed by rank order misarrangement—may adjust which regions have more and less influence in shaping the node’s activity [39**–**41] and thus change the input combinations that drive nodal activation and subsequent behavior. Moreover, alterations in the extent to which overall connection strength is concentrated in a few connections versus distributed evenly across many [24]—indexed by entropy shift—may change the relative magnitudes by which inputs influence nodal activity independent of changes to the rank order of their influence.

It is notable, then, that DS and VS in smokers display distinct forms of connectivity profile misconfiguration. The nature of connectivity profile misconfiguration in the VS suggests that 1) acute abstinence increases the response of VS to marginal frontal input and its overall level of activity, without altering the balance of how much each input shapes its activity, and that 2) achieving nicotine satiety ameliorates these abnormalities. The direction of these results is consistent with prior work that finds FC increases in node-to-node, node-to-network, and network-to-network connections in smokers during short-term abstinence [4, 5, 17, 42, 43], which have also been associated with increases in subjective ratings of withdrawal [5, 44] and worse treatment outcomes [44]. Similarly, we demonstrate that across smokers, higher levels of left nucleus accumbens aggregate divergence following the transition to acute abstinence were associated with greater withdrawal symptomology.

Alternatively, the nature of DS connectivity profile misconfiguration suggests that DS is no more or less responsive to marginal frontal input in smokers compared to non-smokers, but that the relative influence of its frontal inputs on shaping its activity is shuffled—for example, from more motor region influence to more cognitive region influence in the dorsal putamen. This is consistent with prior work noting the increased influence of cognitive frontal regions during abstinence [45, 46]. The stability of DS misconfigurations across smoking states suggests that they are “trait” properties of long-term smokers, which are not normalized even during nicotine satiety. Speculatively, these connectivity profile rank order misarrangements in the DS could reflect alterations associated with the biasing of behavioral control from the goal-directed to the habitual behavior system that is posited to occur in addiction [15, 47]. While the neural basis for this shift has primarily focused on striatal interactions with the dopaminergic midbrain [14], the current findings provide a window for investigating the potential role of frontostriatal interactions in this change.

Notably, we identified one focal striatal area—right caudal ventral putamen—that uniquely displayed both “trait” rank order misarrangement and “state-dependent” aggregate divergence in smokers. While this striatal area constituted only six voxels, the size of functional units in the striatum (~200 μm) [48] is considerably smaller than the size of neuroimaging voxels (3.4 × 3.4 × 4.0 mm in this study). Thus, this cluster likely represents a functionally meaningful area. Here, connections with cognitive frontal areas became stronger than those with motor frontal areas in the connectivity profile as a trait-level misconfiguration. Aggregate divergence during acute abstinence significantly amplified the difference in strength between these classes of connections. Furthermore, when examined across smokers, we found that the interactive magnitude of these two misconfiguration types at this striatal site was significantly associated with dependence severity (i.e., FTND). Yet, there was no relationship with the severity of withdrawal symptomology. These findings suggest that the dual connectivity profile misconfigurations at this site may serve a compensatory function that helps to attenuate dependence during nicotine absence, but via a mechanism other than reduction of aversive withdrawal symptoms. One potential mechanism could be an increase in inhibitory control over habit-driven behavior. This interpretation is consistent with findings that this striatal region is implicated in inhibitory control as well as habit learning and execution [31, 49**–**51]. Misconfiguration of this component of the habit execution circuit during acute abstinence may serve to reduce the propensity for habit-driven smoking behavior despite negative withdrawal symptoms.

Several study experimental design limitations warrant consideration. First, since the subjects in this study were enrolled in a larger smoking cessation clinical trial, the order of sated and abstinent scans was not counterbalanced across subjects, which is often employed to reduce potential confounding effects of scan order. In addition, similar studies often evaluate overnight or 24-h abstinence, and often conduct sated and abstinent scans one or two weeks apart. To increase the intensity of the nicotine withdrawal syndrome (NWS), the present study evaluated 48-h abstinence, and conducted sated and abstinent scans an average of 67 days apart based on individual differences in readiness to enter treatment (median 28 days). Moreover, due to a scanner update during the study, subjects were not all scanned on the same MRI machine, introducing another potential source of between-subject variance. However, the proportion of smokers and non-smokers scanned on each machine did not significantly differ, and no significant differences in connectivity Z-score maps were observed across subjects in relation to which scanner was used.

In sum, we provide evidence that striatal connectivity profiles with frontal cortex are misconfigured in smokers. Prospectively, identified sites of maximal connectivity profile misconfiguration could serve as useful targeting guides for neuromodulation-based therapies and/or as biomarker readouts for treatment efficacy. Further research is warranted to investigate the potential linkages between connectivity profile misconfigurations and cognitive-behavioral functions and the potential significance of laterality effects. It will also be of interest to examine how neuromodulation affects misconfigured connectivity profiles and the degree to which it normalizes them, and how targeting sites identified via a connectivity profile analysis approach impacts treatment outcomes.

## Supplementary information

Supplemental Material
