# Supplementary material for: Misconfigured striatal connectivity profiles in smokers
Source: Neuropsychopharmacology. 2022 Jun 25;47(12):2081–9. doi: 10.1038/s41386-022-01366-6 (PMC9556661; doi:10.1038/s41386-022-01366-6)
Supplement: Supplementary file 1 — Supplemental Material [file 41386_2022_1366_MOESM1_ESM.docx]

**Supplementary Methods**

*Experimental Design*

Upon arrival for their MRI scanning sessions, participants underwent a medical assessment, including a urine test for recent use of illicit drugs (opiates, oxycodone, benzodiazepines, buprenorphine, cocaine, amphetamines/methamphetamines, tetrahydrocannabinol (THC), methadone, phencyclidine, and methlenedioxymethamphetamine (MDMA) and alcohol (Breathalyzer®, Alco-Sensor IV, Intoximeters Incorporated, St. Louis, MO, USA). Positive urine tests were exclusionary for all drugs except THC. For THC, positive urine tests were followed by the Drug Evaluation and Classification neuromotor exam to determine whether the participant was acutely intoxicated[1]. Positive neuromotor exams were exclusionary.

Prior to the first (nicotine sated) MR scanning session, smokers were instructed to smoke *ad lib*; the last cigarette was smoked an average of 48.6 min (SEM=7.69) before MR scanning[2]. Between the sated scan and 48 hours before the second (abstinence scan), a stable smoking pattern was verified by self-report and urine cotinine for all participants. All participants had equivalent cigarettes/day and no quit attempts between the sated and abstinence scans. Smokers were then instructed to abstain from smoking beginning at 48 hours prior to the second MR scanning session, constituting the start of a first quit attempt. Evidence of recent smoking and/or abstinence was assessed by participant self-report of last cigarette and measurement of expired carbon monoxide (CO) (BreathCO, Vitalograph, Lenexa, KS). An expired CO sample < 5 ppm was required to biochemically verify abstinence[3]. In the event of self-reported lapse or CO values > 5 ppm, the second (abstinence) MR scanning session was rescheduled[4]. The treatment protocol that was part of the larger clinical trial was initiated subsequently, and thus did not impact the present study.

*Curating a Matched Normative Sample*

To curate a matched normative sample, we used nearest neighbor matching via the R program MatchIt (<https://cran.r-project.org/web/packages/MatchIt/MatchIt.pdf>) to select healthy, non-smoking or other substance-using subjects (defined below) from the Human Connectome Project (HCP), matched to our empirical sample on age, gender, years of education, and head motion (measured by average framewise displacement on non-censored frames). Individuals from the HCP database were excluded if they reported a family history of schizophrenia, met DSM-IV criteria for alcohol dependence, reported a lifetime history of repeated substance use (>10 instances of cocaine, hallucinogen, opiate, sedatives, or stimulant use, >20 instances of tobacco use or >100 instances of marijuana use), had a urine sample on the day of scanning that was positive for any substance of abuse (cocaine, marijuana, opiates, amphetamine, or methamphetamine), or had a breath sample indicating >0.05 blood alcohol content on the day of scanning. Framewise displacement (FD) for each non-censored frame was computed by applying the following formula[5] to each subject’s timeseries of rigid body parameters:

FDi=∣Δdix∣+∣Δdiy∣+∣Δdiz∣+∣Δαi∣+∣Δβi∣+∣Δγi∣

In all, this procedure resulted in a normative, healthy sample of 79 HCP subjects (the same total size of the empirical sample) age-, gender-, years of education-, and head motion-matched to the empirical sample.

*MRI Data Acquisition*

For resting-state data, 39 oblique axial slices (4-mm thick; 30° to anterior commissure–posterior commissure line) were acquired using a T2*-weighted, single-shot gradient echo, echo planar imaging sequence sensitive to blood oxygenation level-dependent (BOLD) effects (241 volumes; repetition time (TR)=2000 ms; echo time (TE)=27 ms; flip angle (FA)=78°; field of view 220 × 220 mm^2^; image matrix 64 × 64). High-resolution oblique–axial T1-weighted structural images were acquired using a 3D magnetization-prepared rapid gradient-echo (MPRAGE) sequence (TR=1900 ms; TE=3.51 ms; TI=900 ms; FA=9°; voxel size=1 mm^3^).

*Resting-State fMRI Preprocessing*

Results included in this manuscript come from preprocessing performed using FMRIPREP version 20.2.1 [6, 7, RRID:SCR_016216], a Nipype [8, 9, RRID:SCR_002502] based tool. Text in below section was auto-generated via <https://fmriprep.readthedocs.io/en/stable/citing.html>).

Each T1w (T1-weighted) volume was corrected for INU (intensity non-uniformity) using N4BiasFieldCorrection v2.1.0 [10] and skull-stripped using antsBrainExtraction.sh v2.1.0 (using the OASIS template). Brain surfaces were reconstructed using recon-all from FreeSurfer v6.0.1 [11, RRID:SCR_001847], and the brain mask estimated previously was refined with a custom variation of the method to reconcile ANTs-derived and FreeSurfer-derived segmentations of the cortical gray-matter of Mindboggle [26, RRID:SCR_002438]. Spatial normalization to the ICBM 152 Nonlinear Asymmetrical template version 2009c [12, RRID:SCR_008796] was performed through nonlinear registration with the antsRegistration tool of ANTs v2.1.0 [13, RRID:SCR_004757], using brain-extracted versions of both T1w volume and template. Brain tissue segmentation of cerebrospinal fluid (CSF), white-matter (WM) and gray-matter (GM) was performed on the brain-extracted T1w using fast [22] (FSL v5.0.9, RRID:SCR_002823).

Functional data was slice time corrected using 3dTshift from AFNI v16.2.07 [16, RRID:SCR_005927] and motion corrected using mcflirt (FSL v5.0.9 [14]). This was followed by co-registration to the corresponding T1w using boundary-based registration [21] with six degrees of freedom, using bbregister (FreeSurfer v6.0.1). Motion correcting transformations, BOLD-to-T1w transformation and T1w-to-template (MNI) warp were concatenated and applied in a single step using antsApplyTransforms (ANTs v2.1.0) using Lanczos interpolation.

Physiological noise regressors were extracted applying CompCor [23]. Principal components were estimated for the two CompCor variants: temporal (tCompCor) and anatomical (aCompCor). A mask to exclude signal with cortical origin was obtained by eroding the brain mask, ensuring it only contained subcortical structures. Six tCompCor components were then calculated including only the top 5% variable voxels within that subcortical mask. For aCompCor, six components were calculated within the intersection of the subcortical mask and the union of CSF and WM masks calculated in T1w space, after their projection to the native space of each functional run. Frame-wise displacement [24] was calculated for each functional run using the implementation of Nipype.

Many internal operations of FMRIPREP use Nilearn [27, RRID:SCR_001362], principally within the BOLD-processing workflow. For more details of the pipeline see <https://fmriprep.readthedocs.io/en/20.2.1/workflows.html>.

*Regions of Interest*

Regions of interest (ROIs) used for generation of voxel-wise striatal fingerprints were based on the frontal cortical parcellation definitions from the Harvard-Oxford cortical atlas (**Figure S1**) and included: supplementary motor cortex, superior frontal gyrus, subcallosal cortex, precentral gyrus, paracingulate gyrus, middle frontal gyrus, insular cortex, inferior frontal gyrus pars opercularis, inferior frontal gyrus pars triangularis, frontal pole, frontal orbital cortex, frontal operculum cortex, frontal medial cortex, central opercular cortex, and anterior cingulate cortex. The right hemisphere and left hemisphere component of each ROI was parcellated separately. To create sample-specific right and left striatum masks, the union of each subject’s caudate, putamen, and nucleus accumbens FreeSurfer parcellations was taken (separately for each hemisphere), and binary masks were created from the overlap of voxels present in at least 80% of subjects.

Figure S1.

Figure S1. Frontal cortical regions of interest (ROIs) in the Harvard-Oxford atlas used as “targets” in the striatal connectivity profiles.

*Head Motion*

To examine the potential for residual motion to affect FC Z-score maps even after motion censoring, we assessed group/condition differences in subject average FD on non-censored frames. Average subject FD was higher in non-smokers than in smokers in the sated state, t(77)=2.13,p=0.036, and higher in smokers in the abstinent state than in the sated state, t(45)=5.478,p<0.001; average subject FD was not significantly different between non-smokers and smokers in the abstinent state, t(77)=-1.74,p=0.085 (**Supplementary Figure 2a**). The number of frames censored differed significantly between smokers in the sated (mean = 7.54) and abstinent (mean = 13.96) states, t(45) = 3.082, p=0.0035, but not between non-smokers (mean = 12.24) and smokers in the sated state, t(77)=1.907, p=0.060, or smokers in the abstinent state, t(77)=0.542, p=0.589 (**Supplementary Figure 2b**).

Figure S2.

Figure S2. a) Group means for average number of motion-censored frames per subject. B) Groups means for average framewise displacement on non-motion-censored frames per subject.

Given these group differences in motion, we FD-adjusted each subject’s FC Z-score maps. To do this, we first regressed average subject FD on non-censored frames against the FC Z-score map for each ROI across all subjects and computed residual maps for each ROI for each subject, in order to isolate the components of FC not attributable to variance in FD. We then added the group average FC Z-score map for each ROI back onto each subject’s respective ROI residual maps to regain scale. These FD-adjusted Z-score maps served as the inputs to all main analyses.

*Resting-State fMRI Analysis*

Main analyses entailed the computation of three separate connectivity reconfiguration metrics, whose calculation is schematized in **Supplementary Figure 3** and detailed below.

Figure S3.

Figure S3. Illustration of computation procedure for calculating the a) aggregate divergence, b) rank order rearrangement, and c) entropy shift between the connectivity profiles of two voxels with five target ROIs. Numbers in the connectivity profile represent Z-scored functional connectivity between the voxel and each target ROI.

*Aggregate Divergence*

To measure aggregate divergence between the connectivity profile of smokers and non-smokers at each voxel in the striatum, we carried out the following procedure [6] (**Supplementary Figure 4**) to separately compare the average striatal connectivity profiles of non-smokers to 1) the average striatal connectivity profiles of smokers during a nicotine sated state and 2) the average striatal connectivity profiles of smokers following a 48-hour abstinent state.

Figure S4.

Figure S4. Illustration of computation procedure for calculating voxel-wise aggregate divergence between smoker and non-smoker groups from Z-scored functional connectivity maps.

First, we computed voxel-wise striatal Z-score difference maps by taking the absolute value of the difference between the average smoker Z-score map and the average non-smoker Z-score map for each ROI (separately for the sated and abstinent states) – e.g.:

**|Sated Smokers_Z_ - Non-Smokers_Z_|_ROI_**

This produced 30 ROI difference maps for each smoking state. Then, for each state, we took the sum of the 30 ROI difference maps, resulting in final striatal voxel-wise maps whose values represent the Manhattan distance[7] between corticostriatal fingerprints of the average non-smoker and 1) the average sated smoker and 2) the average abstinent smoker – e.g.:


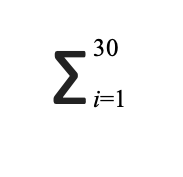
**|Sated Smokers_Z_ - Non-Smokers_Z_|_frontal orbital_** + **|Sated Smokers_Z_ - Non-Smokers_Z_|_frontal medial_ + . . . + |Sated Smokers_Z_ - Non-Smokers_Z_|_insula_**  =

**|Sated Smokers_Z_ - Non-Smokers_Z_|_i_**

*Rank Order Misarrangement*

Instead of constructing striatal voxel fingerprints using the Z-score connectivity values with each frontal cortical ROI, here we used “rank order fingerprints” (**Supplementary Figure 3b**) in which the value of each target ROI was its connectivity strength rank relative to the other ROIs in the fingerprint. As above, this procedure was carried out separately to compare non-smokers to 1) smokers during a nicotine sated state and 2) smokers during a 48-hour abstinent state.

After computing the rank order of each of the 30 cortical ROIs at each striatal voxel in each group based on the group-average ROI Z-score maps, we computed the absolute value of the difference between the rank order of each ROI at each voxel in the smoker group and non-smoker group. The sum of these 30 difference scores was then calculated to represent the “rank order misarrangement” between the groups at each voxel – e.g.:


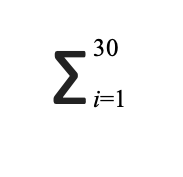
**|Sated Smokers_Rank Order_ - Non-Smokers_Rank Order_ |_frontal orbital_** + **|Sated Smokers_Rank Order_ - Non-Smokers_Rank Order_ |_frontal medial_ + . . . + |Sated Smokers_Rank Order_ - Non-Smokers_Rank Order_ |_insula_**  =

**|Sated Smokers_Rank Order_ - Non-Smokers_Rank Order_ |_i_**

*Entropy Shift*

Using the group-average Z-score connectivity voxel fingerprints, we calculated the entropy – a measure that indexes the uniformity of a distribution – of each voxel’s fingerprint in each group:

where *c_i_* is the magnitude of connectivity with the *i-*th frontal cortical ROI[8]. For each voxel we divided this measure by log_2_30 to normalize it and bind it to the interval [0,1][9]. Higher values of entropy represent connectivity profiles where connectivity strength is more evenly distributed across ROIs. Conversely, lower values of entropy represent connectivity profiles where connectivity strength is more concentrated with one or a few ROIs. The difference in each group’s entropy at each voxel was than computed to represent the “entropy shift” between the groups (**Supplementary Figure 3c**).

*Statistical Significance*

To establish normative distributions for these three metrics, we first randomly split the HCP sample into two groups of sizes equal to those in the empirical sample (i.e., n=33 and n=46). We then calculated the aggregate divergence, rank order misarrangement, and entropy shift between the group average connectivity fingerprints at each striatal voxel in the same manner as for the empirical sample. After storing the resulting distributions, group membership was randomly permuted, and the process was repeated 10,000 times. The data from each permutation was combined to establish a final normative distribution for each of the three metrics. **Supplementary Figure 5** displays the normative distributions and voxel-wise p<0.001 significance thresholds of the three connectivity profile reconfiguration metrics computed from the permutation procedure using the matched HCP sample.

Figure S5.

Figure S5. Normative distributions and voxel-wise p<0.001 thresholds for the connectivity profile reconfiguration metrics.

**Supplemental Results**

*Table S1.* Aggregate Divergence

| ***Groups*** | ***Cluster Region*** | ***Cluster Size (voxels)*** | ***Peak Coordinate*** | ***Peak Manhattan Distance*** |
| --- | --- | --- | --- | --- |
| Abstinent Smokers  vs.  Non-smokers | Right rostral medial striatum | 206 | (3.5, 7.5, -8.5) | 4.65 |
|  | Right caudal lateral striatum | 51 | (33.5, -16.5, -2.5) | 4.27 |
|  | Right caudal lateral striatum | 41 | (21.5, 3.5, -12.5) | 4.45 |
|  | Left rostral medial striatum | 163 | (-14.5, 7.5, 15.5) | 4.18 |
|  | Left caudal lateral striatum | 69 | (-20.5, 3.5, -12.5) | 4.00 |

Table S1. Sites of significant (pFWE < 0.05, cluster size > 7) aggregate divergence in acutely abstinent smokers relative to non-smokers.

*Table S2.* Rank Order Misarrangement

| ***Groups*** | ***Cluster Region*** | ***Cluster Size (voxels)*** | ***Peak Coordinate*** | ***Peak Rank Order Difference*** |
| --- | --- | --- | --- | --- |
| Sated Smokers  vs.  Non-smokers | Right rostral dorsal putamen | *238* | *(23.5, 5.5, 5.5)* | *232* |
|  | Right rostral pole | *66* | *(19.5, 23.5, 5.5)* | *282* |
|  | Right caudal dorsal caudate | *31* | *(15.5, -14.5, 21.5)* | *270* |
|  | Right caudal ventral putamen | *19* | *(29.5, -14.5, -6.5)* | *204* |
|  | Left caudal dorsal putamen | *154* | *(-26.5, -12.5, 11.5)* | *250* |
|  | Left rostral dorsal caudate | *54* | *(-18.5, 19.5, 11.5)* | *242* |
|  | Left rostral dorsal caudate | *12* | *(-14.5, 5.5, 23.5)* | *226* |
| Abstinent Smokers  vs.  Non-smokers | Right rostral dorsal putamen | 94 | *(21.5, 11.5, 7.5)* | *220* |
|  | Right caudal ventral putamen | *20* | *(29.5, -18.5, -0.5)* | *216* |
|  | Right rostral dorsal caudate | *19* | *(17.5, 15.5, 17.5)* | *216* |
|  | Right caudal dorsal putamen | *14* | *(27.5, -10.5, 9.5)* | *206* |
|  | Right caudal dorsal caudate | *14* | *(15.5, -12.5, 23.5)* | *214* |
|  | Right rostral pole | *11* | *(17.5, 25.5, -2.5)* | *220* |
|  | Right caudal dorsal putamen | *9* | *(23.5, -2.5, 11.5)* | *180* |
|  | Left caudal ventral putamen | *49* | *(-30.5, -20.5, 1.5)* | *238* |
|  | Left caudal dorsal putamen | *46* | *(-24.5, 1.5, 13.5)* | *246* |
|  | Left caudal dorsal caudate | *27* | *(-18.5, -4.5, 25.5)* | *20*8 |
|  | Left rostral ventral putamen | *19* | *(-22.5, 5.5, -4.5)* | *190* |
|  | Left rostral pole | *18* | *(-10.5, 21.5, 3.5)* | *218* |

Table S2. Sites of significant (pFWE < 0.05, cluster size > 7) rank order misarrangement in sated smokers and acutely abstinent smokers relative to non-smokers.

*Table S3.* Entropy Shift

| ***Groups*** | ***Cluster Region*** | ***Cluster Size (voxels)*** | ***Peak Coordinate*** | ***Peak Entropy Shift*** |
| --- | --- | --- | --- | --- |
| Sated Smokers  vs.  Non-smokers | Left rostral pole | 9 | (-16.5, 21.5, 7.5) | 0.095 |
| Abstinent Smokers  vs.  Non-smokers | Right nucleus accumbens | 10 | (7.5, 5.5, -10.5) | 0.108 |
|  | Right rostral dorsal caudate | 10 | (9.5, 5.5, 17.5) | 0.093 |
|  | Left caudal dorsal caudate | 9 | (-12.5, 3.5, 21.5) | 0.109 |
|  | Left rostral dorsal caudate | 8 | (-12.5, 7.5, 17.5) | 0.065 |

Table S3. Sites of significant (pFWE < 0.05, cluster size > 7) entropy shift in sated smokers and acutely abstinent smokers relative to non-smokers.

Figure S6.

Figure S6. Clusters of significant connectivity profile reconfiguration within smokers following the transition from nicotine-satiety to 48 hour abstinence.

Figure S7.

Figure S7. Clusters of significant rank order rearrangement in sated smokers (brown) and acutely abstinent smokers (blue) compared to non-smokers. Green clusters indicate areas where significant rank order rearrangement was present during both satiety and acute abstinence.

Figure S8.

Figure S8. Unpacking the interaction between aggregate divergence and rank order rearrangement in the right caudal ventral putamen. The relationship between aggregate divergence and dependence severity (indexed by FTND score) is shown for three representative levels of rank order rearrangement: the mean minus one standard deviation (red), the mean (green), and the mean plus one standard deviation (blue). The model predicts low FTND when both metrics are high (lower right quadrant) and when both metrics are low (lower left quadrant). The model predicts high FTND when one metric is high and when one metric is low (top quadrants).

Right caudal ventral putamen connectivity profile misconfigurations were not significantly associated with withdrawal symptomology during acute abstinence as indexed by the WSWS. However, given prior work implicating nucleus accumbens activity in nicotine withdrawal[10], we examined withdrawal severity in relation to aggregate divergence in the k=11 cluster identified in left nucleus accumbens where this misconfiguration metric increased significantly within smokers between satiety and acute abstinence. We found that, across smokers, greater levels of left nucleus accumbens aggregate divergence during acute abstinence were associated with greater withdrawal symptomology as indexed by WSWS total score during acute abstinence, t(39)=2.209, p=0.033, controlling for age, sex, and education (**Supplementary Figure 9**). Left nucleus accumbens aggregate divergence in acute abstinence was not significantly related to FTND dependence severity, t(39)=0.268, p=0.790.

FTND scores of dependence severity and WSWS scores of withdrawal severity were not significantly related to each other, t(39)=-0.003, p=0.998 (consistent with prior findings of weak univariate relationships between dependence severity and withdrawal[11-13]) nor were the interactive magnitude of aggregate divergence and rank order misarrangement in right caudal ventral putamen and aggregate divergence in left nucleus accumbens, t(37)=0.664, p=0.510.

Figure S9.

Figure S9. Relationship between aggregate divergence in the left nucleus accumbens of acutely abstinent smokers and withdrawal symptomology (indexed by WSWS total score) during acute abstinence.

**References**

1. Heishman, S.J., E.G. Singleton, and D.J. Crouch, Laboratory validation study of drug evaluation and classification program: ethanol, cocaine, and marijuana. J Anal Toxicol, 1996. **20**(6): p. 468-83.

2. Fedota, J.R., et al., Insula demonstrates a non-linear response to varying demand for cognitive control and weaker resting connectivity with the executive control network in smokers. Neuropsychopharmacology, 2016. **41**(10): p. 2557-2565.

3. Javors, M.A., J.P. Hatch, and R.J. Lamb, *Cut‐off levels for breath carbon monoxide as a marker for cigarette smoking.* Addiction, 2005. **100**(2): p. 159-167.

4. Fedota, J.R., et al., *Nicotine abstinence influences the calculation of salience in discrete insular circuits.* Biological Psychiatry: Cognitive Neuroscience and Neuroimaging, 2018. **3**(2): p. 150-159.

5. Power, J.D., et al., Spurious but systematic correlations in functional connectivity MRI networks arise from subject motion. Neuroimage, 2012. 59(3): p. 2142-54.

6. Esteban O, Markiewicz CJ, Blair RW, Moodie CA, Isik AI, Erramuzpe A, Kent JD, Goncalves M, DuPre E, Snyder M, Oya H, Ghosh SS, Wright J, Durnez J, Poldrack RA, Gorgolewski KJ. fMRIPrep: a robust preprocessing pipeline for functional MRI. Nat Meth. 2018; doi:[10.1038/s41592-018-0235-4](https://doi.org/10.1038/s41592-018-0235-4)

7. fMRIPrep Available from: [10.5281/zenodo.852659](https://doi.org/10.5281/zenodo.852659).

8. Gorgolewski K, Burns CD, Madison C, Clark D, Halchenko YO, Waskom ML, Ghosh SS. Nipype: a flexible, lightweight and extensible neuroimaging data processing framework in python. Front Neuroinform. 2011 Aug 22;5(August):13. doi:[10.3389/fninf.2011.00013](https://doi.org/10.3389/fninf.2011.00013).

9. Gorgolewski KJ, Esteban O, Ellis DG, Notter MP, Ziegler E, Johnson H, Hamalainen C, Yvernault B, Burns C, Manhães-Savio A, Jarecka D, Markiewicz CJ, Salo T, Clark D, Waskom M, Wong J, Modat M, Dewey BE, Clark MG, Dayan M, Loney F, Madison C, Gramfort A, Keshavan A, Berleant S, Pinsard B, Goncalves M, Clark D, Cipollini B, Varoquaux G, Wassermann D, Rokem A, Halchenko YO, Forbes J, Moloney B, Malone IB, Hanke M, Mordom D, Buchanan C, Pauli WM, Huntenburg JM, Horea C, Schwartz Y, Tungaraza R, Iqbal S, Kleesiek J, Sikka S, Frohlich C, Kent J, Perez-Guevara M, Watanabe A, Welch D, Cumba C, Ginsburg D, Eshaghi A, Kastman E, Bougacha S, Blair R, Acland B, Gillman A, Schaefer A, Nichols BN, Giavasis S, Erickson D, Correa C, Ghayoor A, Küttner R, Haselgrove C, Zhou D, Craddock RC, Haehn D, Lampe L, Millman J, Lai J, Renfro M, Liu S, Stadler J, Glatard T, Kahn AE, Kong X-Z, Triplett W, Park A, McDermottroe C, Hallquist M, Poldrack R, Perkins LN, Noel M, Gerhard S, Salvatore J, Mertz F, Broderick W, Inati S, Hinds O, Brett M, Durnez J, Tambini A, Rothmei S, Andberg SK, Cooper G, Marina A, Mattfeld A, Urchs S, Sharp P, Matsubara K, Geisler D, Cheung B, Floren A, Nickson T, Pannetier N, Weinstein A, Dubois M, Arias J, Tarbert C, Schlamp K, Jordan K, Liem F, Saase V, Harms R, Khanuja R, Podranski K, Flandin G, Papadopoulos Orfanos D, Schwabacher I, McNamee D, Falkiewicz M, Pellman J, Linkersdörfer J, Varada J, Pérez-García F, Davison A, Shachnev D, Ghosh S. Nipype: a flexible, lightweight and extensible neuroimaging data processing framework in Python. 2017. doi:[10.5281/zenodo.581704](https://doi.org/10.5281/zenodo.581704).

10. Tustison NJ, Avants BB, Cook PA, Zheng Y, Egan A, Yushkevich PA, Gee JC. N4ITK: improved N3 bias correction. IEEE Trans Med Imaging. 2010 Jun;29(6):1310–20. doi:[10.1109/TMI.2010.2046908](https://doi.org/10.1109/TMI.2010.2046908).

11. Dale A, Fischl B, Sereno MI. Cortical Surface-Based Analysis: I. Segmentation and Surface Reconstruction. Neuroimage. 1999;9(2):179–94. doi:[10.1006/nimg.1998.0395](https://doi.org/10.1006/nimg.1998.0395).

12. Fonov VS, Evans AC, McKinstry RC, Almli CR, Collins DL. Unbiased nonlinear average age-appropriate brain templates from birth to adulthood. NeuroImage; Amsterdam. 2009 Jul 1;47:S102. doi:[10.1016/S1053-8119(09)70884-5](https://doi.org/10.1016/S1053-8119(09)70884-5).

13. Avants BB, Epstein CL, Grossman M, Gee JC. Symmetric diffeomorphic image registration with cross-correlation: evaluating automated labeling of elderly and neurodegenerative brain. Med Image Anal. 2008 Feb;12(1):26–41. doi:[10.1016/j.media.2007.06.004](https://doi.org/10.1016/j.media.2007.06.004).

14. Jenkinson M, Bannister P, Brady M, Smith S. Improved optimization for the robust and accurate linear registration and motion correction of brain images. Neuroimage. 2002 Oct;17(2):825–41. doi:[10.1006/nimg.2002.1132](https://doi.org/10.1006/nimg.2002.1132).

15. Andersson JLR, Skare S, Ashburner J. How to correct susceptibility distortions in spin-echo echo-planar images: application to diffusion tensor imaging. Neuroimage. 2003 Oct;20(2):870–88. doi:[10.1016/S1053-8119(03)00336-7](https://doi.org/10.1016/S1053-8119(03)00336-7).

16. Cox RW. AFNI: software for analysis and visualization of functional magnetic resonance neuroimages. Comput Biomed Res. 1996 Jun;29(3):162–73. doi:[10.1006/cbmr.1996.0014](https://doi.org/10.1006/cbmr.1996.0014).

17. Jenkinson M. Fast, automated, N-dimensional phase-unwrapping algorithm. Magn Reson Med. 2003 Jan;49(1):193–7. doi:[10.1002/mrm.10354](https://doi.org/10.1002/mrm.10354).

18. Huntenburg JM. Evaluating nonlinear coregistration of BOLD EPI and T1w images. Freie Universität Berlin; 2014. Available from: <http://hdl.handle.net/11858/00-001M-0000-002B-1CB5-A>.

19. Wang S, Peterson DJ, Gatenby JC, Li W, Grabowski TJ, Madhyastha TM. Evaluation of Field Map and Nonlinear Registration Methods for Correction of Susceptibility Artifacts in Diffusion MRI. Front Neuroinform. 2017 [cited 2017 Feb 21];11. doi:[10.3389/fninf.2017.00017](https://doi.org/10.3389/fninf.2017.00017).

20. Treiber JM, White NS, Steed TC, Bartsch H, Holland D, Farid N, McDonald CR, Carter BS, Dale AM, Chen CC. Characterization and Correction of Geometric Distortions in 814 Diffusion Weighted Images. PLoS One. 2016 Mar 30;11(3):e0152472. doi:[10.1371/journal.pone.0152472](https://doi.org/10.1371/journal.pone.0152472).

21. Greve DN, Fischl B. Accurate and robust brain image alignment using boundary-based registration. Neuroimage. 2009 Oct;48(1):63–72. doi:[10.1016/j.neuroimage.2009.06.060](https://doi.org/10.1016/j.neuroimage.2009.06.060).

22. Zhang Y, Brady M, Smith S. Segmentation of brain MR images through a hidden Markov random field model and the expectation-maximization algorithm. IEEE Trans Med Imaging. 2001 Jan;20(1):45–57. doi:[10.1109/42.906424](https://doi.org/10.1109/42.906424).

23. Behzadi Y, Restom K, Liau J, Liu TT. A component based noise correction method (CompCor) for BOLD and perfusion based fMRI. Neuroimage. 2007 Aug 1;37(1):90–101. doi:[10.1016/j.neuroimage.2007.04.042](https://doi.org/10.1016/j.neuroimage.2007.04.042).

24. Power JD, Mitra A, Laumann TO, Snyder AZ, Schlaggar BL, Petersen SE. Methods to detect, characterize, and remove motion artifact in resting state fMRI. Neuroimage. 2013 Aug 29;84:320–41. doi:[10.1016/j.neuroimage.2013.08.048](https://doi.org/10.1016/j.neuroimage.2013.08.048).

25. Pruim RHR, Mennes M, van Rooij D, Llera A, Buitelaar JK, Beckmann CF. ICA-AROMA: A robust ICA-based strategy for removing motion artifacts from fMRI data. Neuroimage. 2015 May 15;112:267–77. doi:[10.1016/j.neuroimage.2015.02.064](https://doi.org/10.1016/j.neuroimage.2015.02.064).

26. Klein A, Ghosh SS, Bao FS, Giard J, Häme Y, Stavsky E, et al. Mindboggling morphometry of human brains. PLoS Comput Biol 13(2): e1005350. 2017. doi:[10.1371/journal.pcbi.1005350](https://doi.org/10.1371/journal.pcbi.1005350).

27. Abraham A, Pedregosa F, Eickenberg M, Gervais P, Mueller A, Kossaifi J, Gramfort A, Thirion B, Varoquaux G. Machine learning for neuroimaging with scikit-learn. Front in Neuroinf 8:14. 2014. doi:[10.3389/fninf.2014.00014](https://doi.org/10.3389/fninf.2014.00014).
